# A Mining Accident

**Published:** 1900-05-05

**Authors:** 


### A MINING ACCIDENT.

A very extraordinary occurrence is reported in the *British Medical Journal* by Mr. Ernest V. Eames. It happened in descending a colliery shaft. A cage in which 18 men were being lowered into the pit, 200 yards deep, ran away—that is, the engineman lost control of his engine when the cage was about 60 yards from the bottom. Consequently the men were precipitated down the shaft at a terrific speed until the cage was brought to a sudden stand by impact with the planks placed across the "sump hole" at the bottom. The men during their descent are said habitually to stand in a rather stooping position, with the legs straight and the head and shoulders rather bent. Now of the 18 men five suffered dislocation of the knee, all in the same direction, the head of the tibia and the fibula being carried forwards and shot up on the anterior surface of the femur. In three of the cases the skin was split across the back of the joint, but without opening the joint cavity, and in one there was an oblique fracture of the tibia as well as dislocation of the knee. In all the cases but one a good result was obtained, the men being at work under five months from the date of the accident, with practically sound knees, of the same appearance as before, and of equal usefulness. The case in which a bad result followed was the one which was complicated with fracture of the tibia. In this case the man remains with a flat joint, and he also has paralysis of the extensor muscles of the foot. The form of dislocation here met with is sufficiently uncommon, but its occurrence in five men at the same time seems quite unique.

